# Autoimmune Gastritis and Hypochlorhydria: Known Concepts from a New Perspective

**DOI:** 10.3390/ijms25136818

**Published:** 2024-06-21

**Authors:** Marica Vavallo, Sophia Cingolani, Giulio Cozza, Francesco P. Schiavone, Ludovica Dottori, Carla Palumbo, Edith Lahner

**Affiliations:** Gastroenterology Unit, Sant’Andrea University Hospital, Department of Medical-Surgical Sciences and Translational Medicine, Sapienza University of Rome, 00189 Rome, Italygiulio.cozza@uniroma1.it (G.C.); schiavone.1794884@studenti.uniroma1.it (F.P.S.);

**Keywords:** autoimmune gastritis, cobalamin (vitamin B12) deficiency, gastric cancer, gastric microbiota, gastrin, iron deficiency anemia, pernicious anemia, type 1 gastric neuroendocrine tumors

## Abstract

Autoimmune atrophic gastritis is an immune-mediated disease resulting in autoimmune destruction of the specialized acid-producing gastric parietal cells. As a consequence, in autoimmune atrophic gastritis, gastric acid secretion is irreversibly impaired, and the resulting hypochlorhydria leads to the main clinical manifestations and is linked, directly or indirectly, to the long-term neoplastic complications of this disease. In the last few years, autoimmune atrophic gastritis has gained growing interest leading to the acquisition of new knowledge on different aspects of this disorder. Although reliable serological biomarkers are available and gastrointestinal endoscopy techniques have substantially evolved, the diagnosis of autoimmune atrophic gastritis is still affected by a considerable delay and relies on histopathological assessment of gastric biopsies. One of the reasons for the diagnostic delay is that the clinical presentations of autoimmune atrophic gastritis giving rise to clinical suspicion are very different, ranging from hematological to neurological–psychiatric up to gastrointestinal and less commonly to gynecological–obstetric symptoms or signs. Therefore, patients with autoimmune atrophic gastritis often seek advice from physicians of other medical specialties than gastroenterologists, thus underlining the need for increased awareness of this disease in a broad medical and scientific community.

## 1. Introduction

Autoimmune atrophic gastritis (AAG) is a not-self-limiting, immune-mediated disorder resulting in self-destruction of the highly specialized acid-producing cells of the corpus oxyntic mucosa, the final target of the autoimmune reaction mediated by cytotoxic T cells. This disease is probably much more frequent than currently diagnosed, and one of the reasons is that there are multiple clinical presentations ranging from hematological to neurological–psychiatric up to gastrointestinal and, less commonly, to gynecological–obstetric manifestations. AAG is also linked to an increased risk of type 1 gastric neuroendocrine tumors (t1-gNETs) and, albeit still debated, gastric cancer. T1-gNETs are generally benign with a typically indolent course and a negligible risk of metastasis.

The main driver of clinical manifestations and neoplastic complications in AAG is the altered gastric microenvironment as a consequence of the atrophy of the oxyntic mucosa leading to reduced secretion of gastric acid and thus to hypo/achlorhydria and hypergastrinemia. The current review provides modern insights into different aspects of AAG, from epidemiology and pathogenesis to clinical manifestations, the origin of impaired gastric acid secretion, gastric microbiota alterations and the risk of neoplastic complications.

## 2. Epidemiological, Pathogenetic, Clinical and Diagnostic Issues of Autoimmune Atrophic Gastritis

Autoimmune atrophic gastritis (AAG) is a chronic autoimmune disorder resulting in loss of the oxyntic glands of the gastric body, atrophy and intestinal and/or pseudo-pyloric metaplasia [1,2,3,4,5,6]. The overall prevalence of the disease is estimated to be around 0.5–4.5% [7]. Higher prevalence values, up to 8–20%, are reported in studies that consider serological diagnosis [8]. The prevalence of this disease is higher in women (ratio 2.25:1) [9] and in patients with other autoimmune diseases, such as type I diabetes mellitus and Hashimoto thyroiditis (thyrogastric syndrome) [7,10,11,12,13]. According to some authors [10,14], prevalence is probably underestimated due to the lack of symptoms in the early stages of the disease. 

The pathogenesis of AAG remains unclear, and two hypotheses have been proposed: (i) a pure autoimmune disease, and (ii) an immune-mediated response to *Helicobacter pylori* (Hp) infection, possibly caused by a mechanism of molecular mimicry between Hp antigens and proton pump H+/K+ ATPase of the parietal cells in the gastric body [15,16,17,18,19,20]. This membrane protein is recognized by parietal cell autoantibodies (PCAs) [21,22]. 

However, these antibodies do not have a pathogenetic role, and damage is induced by self-reactive T cells [23,24,25]. 

D’Elios et al. tested the in vitro reactivity of CD4+ and CD8+ lymphocytes isolated from the antrum and corpus biopsies of patients with AAG, showing that CD4+ cells were the only ones that react to their antigens under MHC class II-restricted conditions. The majority of H+, K+ ATPase-specific clones produced IFN-γ but neither IL-4 nor IL-5 (Th1 cytokine profile), and all of these single clones, but none of the Hp-specific T cells, produced TNF-α. Thus, lymphocytes can self-sustain autoimmune response by producing IFN-γ. Indeed, IFN-γ seems to be able to stimulate gastric cells to express MHC class II molecules and other costimulatory molecules (e.g., CD80, CD86). By the end, Th1 cells appear to trigger apoptosis in parietal cells through both perforins and Fas–Fas ligand interaction, and they also seem to activate B cells to produce IgM, IgG and IgA found in sera [24]. Other cells (e.g., Th2, Th17, macrophages, epithelial cells and their derived mediators) and cytokines (e.g., IL4, IL9, IL12 and IL17) may play a role in the pathogenesis of AAG since many of the mechanisms of immune damage are yet to be discovered [23]. 

Hp involvement in AAG can be suggested by common features between AAG and Hp-related multifocal chronic atrophic gastritis. First of all, Hp-specific autoantibodies cross-react with human gastric mucosa. Secondly, Hp antibodies have already been observed in patients with AAG, and PCAs can be expressed, in the same proportions, by patients with AAG and patients with Hp-related chronic atrophic gastritis [10,18,19].

In histology, the early inflammatory phase is characterized by a rich and heterogeneous inflammatory infiltrate including a prominent eosinophilic component. A later atrophic stage is proven by loss of the oxyntic glands, micro-scars, atrophy, pseudo-pyloric and/or intestinal metaplasia. Pseudo-pyloric metaplasia, also known as spasmolytic polypeptide-expressing metaplasia (SPEM), refers to the antralization of the gastric body. Intestinal metaplasia is characterized by the presence of intestinal goblet cells capable of expressing CDX2 [10,26,27,28].

The disappearance of acid-secreting mucosa determines hypo- or achlorhydria, hypergastrinemia, and hyperplasia of enterochromaffin-like (ECL) cells, both linear and nodular, which over time may give rise to gastric neuroendocrine dysplasia and eventually tumors (t1-gNETs) [10]. 

Achlorhydria and intrinsic factor deficit lead to iron deficiency anemia (IDA) and pernicious anemia (PA), respectively. Intrinsic factor is necessary for the absorption of cobalamin, which plays an important role in myelopoiesis and neurogenesis. Therefore, the earliest symptom of cobalamin (vitamin B_12_) deficiency is paresthesia of the hands and distal extremities, present in up to 70% of patients [27,29]. Other neurological symptoms (numbness, altered proprioception, ataxia, behavioral and mood disorders, and psychosis) are caused by neuronal demyelination and degeneration, and if not promptly treated, they may be irreversible [6,11,30,31]. Furthermore, vitamin B_12_ deficiency causes hyperhomocysteinemia, eventually leading to thrombosis [32,33,34], infertility, abortion [10,35], and glossitis [36]. For a long time, PA has been used as a synonym of AAG. PA is the most known hematological presentation of disease and is characterized by megakaryocytes (MCV > 100 fl), anisocytosis and in some cases pancytopenia. Moreover, PA and IDA may concurrently cause dysmorphic anemia with normal MCV and anisocytosis [37]. Symptoms of anemia, of all kinds, are predominantly fatigue, dyspnea, peripheral edema, and, in some cases, heart failure [10,38,39]. Unfortunately, anemic patients (without gastrointestinal bleeding) often are not referred to gastroenterologists, and anemia is treated without further investigation, causing a great diagnostic delay [10,11,35].

Gastrointestinal symptoms of AAG are supposed to result from gastric–duodenal bacterial overgrowth and reduced gastric motility due to achlorhydria. Dyspepsia is the earliest gastrointestinal symptom [40,41], and paradoxically, when it is the only presentation symptom, it is the one associated with the highest diagnostic delay (>24 months) [11]. The most commonly reported dyspeptic symptoms are early fullness, post-prandial bloating, and epigastric discomfort. Other reported gastrointestinal symptoms of AAG are nausea, weight loss [6,11,41,42] and even gastro-esophageal reflux; studies suggest not excluding the diagnosis of AAG in patients who report reflux-like symptoms [38,43]. Non-acid reflux has been identified as a cause of such symptoms in patients with AAG [10].

As previously said, PCAs are the hallmark of AAG. PCA dosage is useful for screening patients at risk of AAG, such as those with other autoimmune diseases and/or symptoms. Intrinsic factor autoantibodies (IFAs) may also be positive in AAG patients. However, IFAs are highly specific but poorly sensitive [10,44]. Several studies suggested that any type of atrophy can be diagnosed by dosing serum gastrin and pepsinogen (Pg) I and II and determining the PgI/II ratio [45,46,47,48]. In AAG patients, the PgI/PgII ratio is low because PgI is no longer produced by the oxyntic glands of the gastric body. A recent study showed a high diagnostic reliability of serum PgI for diagnosing AAG with a sensitivity and specificity of 92.7% and 99.1%, respectively, and for diagnosing CLEIA with a sensitivity and specificity of 90.5% and 98.2%, respectively [49]. Serum PgI levels also correlate with the severity of the atrophic damage. A great advantage of the so-called “serological gastric biopsy” is that, reflecting the pathophysiology of the whole stomach, it is not affected by sampling error. Moreover, dosing IgG against Hp is useful to assess exposure to the bacterium. Checchi S et al. [50] suggested that, regardless of Hp infection, serum ghrelin is more sensitive and specific than serological gastric biopsy, but further validation is needed. 

However, 2.5–9% of healthy individuals have positivity against PCAs, and a subgroup of patients with AAG is seronegative for PCAs (especially elderly patients) [51] indicating that seropositivity alone is not enough for the diagnosis of AAG [52,53]. However, recent studies show that seropositivity against PCAs in subjects with a healthy gastric mucosa might represent a very early stage of “potential” AAG that may evolve to overt AAG, thus probably meriting surveillance [54]. According to what is mentioned above, gastroscopy with biopsies, according to the updated Sydney system [53], is necessary for confirming the diagnosis of AAG.

## 3. Autoimmune Atrophic Gastritis and Hypochlorhydria: Why Do They Occur and How Can They Be Assessed?

The stomach produces gastric juice (nearly 1.5–3.5 liters per day), which is an acidic liquid (pH < 2) containing a high concentration of hydrochloric acid (HCl) actively secreted by oxyntic mucosa, lipase, and pepsin [55].

Hypochlorhydria occurs in AAG due to the destruction of the oxyntic mucosa in the stomach. The oxyntic mucosa contains chief cells, mucous neck cells, enterochromaffin-like (ECL) cells and parietal cells. Parietal cells have two main functions: hydrochloric acid secretion and intrinsic factor production. They are crucial for generating the stomach’s acidic environment, and their function is tightly regulated by both hormonal and neural stimulation. Histamine, secreted by ECL cells of the corpus and fundus, and gastrin, secreted by gastrin-producing cells in the antrum, can stimulate the secretion of hydrochloric acid and intrinsic factor by parietal cells. 

On their secretory membrane, parietal cells have a proton pump, the H+/K+ ATPase, composed of two alfa subunits and two beta subunits, which guarantees the maintenance of an acid gastric environment by transporting H+ protons and K+ against their concentration gradient, using ATP hydrolysis, towards the luminal side [52,56]. This ATPase is finely regulated by both endocrine and neuronal mechanisms, including vagal stimulation and acetylcholine release, stimulating histamine release by ECL cells and gastrin release by antral G cells, and inhibiting somatostatin release by D cells [57,58,59,60]. All these fine mechanisms are finalized in the maintenance of an acid gastric pH that is fundamental for digestion processes, micronutrient absorption, and maintenance of the epithelial barrier. As explained before, this proton pump is the major target antigen recognized by PCAs [17], and it targets α-subunits and β-subunits of the pump [22]. Activated autoreactive T cells produce pro-inflammatory cytokines, enhancing immune response and favoring parietal cell apoptosis through Fas–Fas ligand (FasL) and perforin–granzyme mechanisms, ultimately leading to oxyntic mucosa atrophy and consequent decrease in HCl production ranging over time from impaired (hypochlorhydria) to absent acid secretion (achlorhydria) [10]. 

When AAG is clinically suspected, non-invasive serological tests can be performed before the patient undergoes a gastroscopy with biopsy, necessary for the final diagnosis of AAG. These markers are gastrin and pepsinogens, combined with PCAs and IFAs. As mentioned before, pepsinogen I is secreted by chief cells of the oxyntic mucosa, while pepsinogen II is produced by the pyloric glands, leading to decreased pepsinogen I in AAG and decreased pepsinogen II in antral atrophy. Gastrin is a hormone secreted by gastrin-producing cells in the antrum. Its primary function is to stimulate the secretion of gastric acid by the parietal cells. With the progressive destruction of parietal cells, hydrochloric acid and intrinsic factor production decreases, resulting in hyperplasia of gastrin-producing cells and ECL cells and hypergastrinemia. Over 90% of human gastrins are α-amidated and bioactive, with the majority being gastrin-17, followed by gastrin-34, and smaller amounts of gastrin-71, gastrin-52, gastrin-14 and gastrin-6 [61].

RIA assays that target the active site of gastrin peptides (the C-terminal tetrapeptide amide sequence) are becoming less available, and, nowadays, they have been largely replaced by commercial kits, primarily ELISA-based, which often measure only one form of gastrin, typically gastrin-17. This is a limitation that can lead to false-negative results, while false positives may occur due to interactions with plasma proteins or O-sulfated proteins, reducing the diagnostic reliability of hypergastrinemia. Many factors like antral distension, vagal stimulation from sensory activities, undigested proteins, alcohol, hypoglycemia, caffeine and high calcium levels can trigger gastrin release, while gastric acid and inhibitory hormones like secretin, GIP, VIP, glucagon, calcitonin and somatostatin can suppress gastrin release [62]. Limitations in testing gastrin levels are due to gastrin concentrations also being influenced by proton pump inhibitors (PPIs), commonly prescribed drugs, that inhibit the gastric proton pump. This inhibition leads to compensatory increased secretion of gastrin. Thus, PPI treatment should be withdrawn at least 14–20 days before gastrin assessment to avoid false positives. Another common cause of elevated gastrin levels is Hp infection, likely due to the activation of pro-secretory products from inflammation. To accurately measure plasma gastrin levels, it is important to suspend PPI treatment before blood collection to obtain a true reading, and the possibility of Hp infection should be considered when high gastrin levels are detected.

A systematic review evaluated the diagnostic accuracy of combining pepsinogen, gastrin-17 and anti-Hp antibody serum tests for diagnosing atrophic gastritis. Without stimulation tests, basal gastrin-17 alone showed 62% sensitivity and 96.1% specificity for diagnosing atrophic gastritis. Sensitivity and specificity were higher in patients not taking PPIs compared to those chronically using PPIs [63]. The performance of serum panel test assessed on 20 studies with a total of 4241 patients for atrophic gastritis diagnosis regardless of the site in the stomach gave a summary sensitivity and specificity of 74.7% and 95.6%, respectively, while the summary sensitivity and specificity for the diagnosis of corpus-limited atrophic gastritis assessed in seven studies were 70.4% and 98.4%, respectively [63]. 

Gastric juice testing in AAG can be useful to confirm low gastric acid secretion. The traditional basal and pentagastrin-stimulated acid secretion investigation is complex and invasive and has become obsolete in clinical practice. A real-time device for analysis of gastric acidity has been developed to analyze the pH of gastric juice aspirated during upper gastrointestinal endoscopy, and ammonium concentration to assess for Hp infection, and communicate the result during examination [64]. It needs 3 mL of gastric juice, and it measures the pH value in 15 s and ammonium concentration for the detection of Hp infection generally in 60 s [65]. 

From a 2023 review by Zullo et al. that considered data from 11 studies [64], it emerged that standard biopsy sampling in the stomach could be eventually avoided in several patients with normally appearing gastric mucosa but without Hp infection or gastric atrophy in this real-time analysis (risk of false negative: 3–4 patients every 100). The values of sensitivity, specificity, positive predictive value, negative predictive value, accuracy, positive likelihood ratio and negative likelihood ratio for suspecting diffuse atrophic gastritis were 83%, 92%, 58%, 97%,91%, 9.9 and 0.2. respectively. This device, by analyzing the gastric juice, can help to improve the quality of gastroscopy, reducing biopsies if useless and also alerting the endoscopist to obtain the standard antral and gastric body mucosa specimens in those patients with positivity for at least one finding [64].

## 4. AAG and Hypochlorhydria: What Are the Consequences on Iron Absorption and Clinical Management?

As mentioned above, autoreactive T cells, both T-helper 1 and cytotoxic T, are thought to be the real effectors of parietal cell damage in AAG, eliciting and actualizing the immune response against the oxyntic mucosa [2,10], leading to impaired acid secretion.

In particular, HCl secretion mediated by H^+^/K^+^-ATPase, together with ascorbic acid, plays a crucial role in the reduction of non-heme iron from the ferric form to the ferrous form, which is likely the only one to be transported into the enterocytes [66,67]. Moreover, ascorbic acid chelates ferric iron, preventing its precipitation and polymerization, and pH < 3 guarantees the solubility of ferric iron [67,68]. Nonetheless, heme iron is directly absorbed by small intestine enterocytes and therefore less affected by these mechanisms, but it accounts just for 5–10% of the human iron dietary intake, with non-heme iron being responsible for 80% of oral iron intake [69].

On this basis, the progressive destruction of parietal cells due to the Th1 immune response is not only the main histopathological hallmark of AAG but also directly linked to one of its main clinical manifestations, namely IDA [10,70]. The progressive destruction of parietal cells means a progressive loss of acid secretion into the stomach, inevitably leading to hypochlorhydria and, progressively, achlorhydria [10]. This increase in gastric pH determines a lack of dietary iron dissolution and reduction, causing its malabsorption [10,57,58,59,60,66,67,68,69]. As iron liver storages are dissipated in a few months in case of reduced absorption, IDA is frequently the earliest clinical manifestation of AAG, being present in up to 50% of patients and leading to AAG diagnosis in many cases [2,10,70,71,72].

IDA of AAG is typically a chronic anemia, progressively arising, more frequent in females, and responsible for weakness, headache, physical and cognitive performance reduction, having an overall bad impact on quality of life and enhancing the risk for cardiac events and mortality [72,73,74].

Moreover, IDA in AAG patients is more insidious to treat, due to an intrinsic impairment of iron absorption that leads to poor efficacy of oral therapy [72]. Indeed, several studies have shown IDA refractoriness to oral iron therapy in cases of AAG: Annibale et al. showed that out of 71 patients with oral iron refractory IDA, 19 had chronic atrophic gastritis, and 6 of these cases were Hp-negative [75]. Hershko et al. found up to 70% oral iron refractoriness in patients with PCA positivity or Hp infection [76]. More recently, Rogez et al. demonstrated the inefficacy of a 3-month course of oral iron therapy in patients with PA and IDA [77].

As strengthened in the study by Hershko et al., Hp infection itself can also be a cause of oral iron therapy refractoriness [76]; moreover, Hp can be associated with AAG and is supposed to be a possible trigger for autoimmune activation due to molecular mimicry between Hp epitopes and parietal cell H^+^/K^+^-ATPase; thus, it can be a reasonable clinical strategy in AAG patients to accurately look for and promptly treat Hp infection to improve iron absorption, removing at least one cause of iron malabsorption.

Not to be underestimated, AAG is a chronic condition, thus requiring iron supplementation for an indefinite time, and oral iron therapy unfortunately is poorly tolerated by patients because of its common and unpleasant side effects [78], frequently leading to low adherence to treatment.

For these reasons, clinical management of patients with IDA in AAG frequently includes the use of intravenous iron treatment [72,79]. Nowadays, a variety of intravenous iron formulations are available, and the more recent ones have a low risk of adverse reactions and events; they include low-molecular-weight dextrane, iron isomaltoside, ferumoxytol and ferric carboxymaltose (FCM). This last one has been specifically tested in patients with corpus atrophic gastritis, both autoimmune and not, and has been shown to be safe and effective with a good performance in increasing hemoglobin levels and avoiding anemia recurrence for nearly 24 months [72]. Only a few AAG cases require blood transfusions, generally patients with severe anemia, in whom AAG is frequently misdiagnosed because of its chronic nature, and with comorbidities, mainly cardiovascular ones. 

Given the chronicity of AAG, in the clinical management of these patients, it is very important to periodically monitor blood cell count, ferritin, transferrin and iron levels to prevent anemia recurrence and promptly offer treatment with iron supplementation [2,10,72,79].

## 5. AAG and Hypochlorhydria: What Are the Consequences on Cobalamin Absorption and Clinical Management?

The atrophy of parietal cells, responsible for HCl and intrinsic factor (IF) secretion, as well as the presence of IFAs, both occurring in AAG, determine a reduced absorption of cobalamin (vitamin B_12_) [80]. Specifically, HCl plays a crucial role in this process by splitting vitamin B_12_ from other food proteins permitting its bond with haptocorrin, also known as cobalophilin, which is a glycoprotein secreted by salivary glands that protects vitamin B_12_ from degradation inside the stomach. In the duodenum, the action of pancreatic enzymes and the increase in pH induce the cleavage of the haptoglobin–vitamin B_12_ complex, thus allowing vitamin B_12_ to bind with IF, a glycoprotein that mediates its internalization into the enterocytes of the distal jejunum where IF is recycled while vitamin B_12_ passes into circulation through the MDR1 transporter. Once in the blood, vitamin B_12_ will be bonded by different carrier proteins such as transcobalamin 1 and 2 [14,81].

It is therefore clear that the reduced secretion of one or both of these factors, as well as the action of IFAs, which inhibit IF function, can result in reduced absorption of vitamin B_12_, which, in addition, cannot be synthesized de novo by the human body.

Apart from AAG, there are other conditions, both physiological and pathological, that can lead to cobalamin deficiency. These include chronic use of anti-secretory drugs such as proton pump inhibitors and H_2_ blockers; a reduced dietary intake of cobalamin, as in the case of a vegetarian or vegan diet; and a condition of gastritis caused by chronic Hp infection affecting the corpus–fundus mucosa causing hypochlorhydria [82,83]. In this circumstance, the successful eradication of Hp and the resulting healing of gastric oxyntic mucosa can determine the resolution of cobalamin deficiency [84]. 

The main clinical manifestations of vitamin B_12_ deficiency are the onset of typically megaloblastic anemia, called PA; increased blood levels of homocysteine; demyelination of peripheral nerves; and infertility. The symptoms most frequently reported are asthenia; paraesthesia affecting especially the extremities; irritability; and, in some cases, cognitive impairment. In addition, there is an increased risk of thrombotic events caused by hyperhomocysteinemia [35,37,85]. It follows how, in case of its deficiency, it is important to promptly resume iatrogenic supplementation of vitamin B_12_ to reverse these manifestations that, in the long term, may represent a risk to the patient’s life.

Although, as of today, there is no standardized protocol for vitamin B_12_ supplementation in patients with AAG and PA, an authoritative opinion suggests that an efficient therapy consists of the administration of vials containing a more stable form of cobalamin, namely cyanocobalamin, intramuscularly [84]. 

In case of severe neurological symptoms, high-dose treatment based on daily intramuscular injections for a week followed by weekly injections until improvement of symptoms has been suggested [14]. However, considering the wide interpersonal variability in response, a clinical and laboratory follow-up is necessary in order to define the most appropriate dosage for each patient. In our center, to treat vitamin B_12_ deficiency in AAG, we administer a treatment scheme consisting of an initial loading dose of 15,000 µg of cyanocobalamin intramuscularly, divided into three administrations to be given at 5-day intervals followed by a maintenance phase during which 5000 µg of cyanocobalamin is administered intramuscularly every 3 months. However, it has been observed that not all patients respond adequately to this treatment schedule. In cases of inadequate response to this treatment scheme, we usually modify the dosage of cyanocobalamin by decreasing the interval between administrations to one or two months, depending on the patient’s response. Recently, we conducted a study on 190 AAG patients to assess the factors that could predict each patient’s need for cyanocobalamin to manage them for the best from the beginning of therapy. Of the 190 AAG patients, 31.6% did not reverse cobalamin deficiency with the standard protocol of 5000 µg of cyanocobalamin administered intramuscularly every 3 months, but needed to be shifted to 5000 µg of cyanocobalamin administered intramuscularly every 2 months. Our results underlined how male gender, a severe grade of intestinal metaplasia at diagnostic endoscopy and an increased MCV value at diagnosis were significantly associated with the need for an increased dosage of cyanocobalamin to recover from its deficiency [86].

The main possible side effects of intramuscular cyanocobalamin administration are represented by allergic reactions with cutaneous manifestations such as rash and/or pruritus, fever and hypokalemia [87]. Other therapeutic options that could represent valid alternatives due to ease of administration are oral or sublingual vitamin B_12_ intake. However, their efficacy in the long-term treatment of cobalamin deficiency in AAG patients has not been investigated so far.

## 6. AAG and Hypochlorhydria: What Are the Consequences on the Gastric Microbiota?

The stomach was originally considered a sterile environment, unsuitable for bacterial growth, because of the presence of hydrochloric acid, its proteolytic action and the production of nitric oxide at the salivary level. The discovery of Hp, later classified as a class I carcinogen, led to a re-evaluation of these considerations and to a gradual increase in interest in the study of gastric bacteria; thus, the first studies aimed at investigating the presence of gastric bacterial forms based on the culture method were conducted. However, these studies had limitations, the most important of which lay in the fact that most (80%) of microbial species were not culturable; other limits were the type of gastric specimen that was often limited to luminal content rather than bacteria directly associated with the mucosa, the different acidity of the gastric environment at the time of culture and the method of culture, the difficulty in obtaining by biopsies or the collection of a gastric juice sample that was representative of the whole gastric microbiota, and the contamination of gastric microorganisms by those from the oral cavity or throat. These limitations led to the development of different techniques, such as DNA genome sequencing and molecular microbiome analysis, which also allowed selective detection of live bacteria, which was not possible with culture examination [88].

A 2008 study using the DNA sequencing method aimed to characterize the gastric microbiome and showed that the diversity of the bacterial composition of the stomachs of healthy patients was higher compared to patients with Hp infection [88]. Other studies have shown how, in cases of Hp infection, it represents between 40% and 95% of the gastric microbiome [89]. After the eradication of the bacterium by antibiotic therapy, the restoration of bacterial abundance similar to that of patients who never contracted the infection can be achieved, showing how the bacterium-induced condition is reversible [88]. Today we know that the gastric microbiota, in addition to Hp, comprises about 10^3^–10^4^ CFU [90]. The most frequent bacterial phyla in the healthy stomach are Proteobacteria, Firmicutes, Bacteroidetes, Actinobacteria and Fusobacteria; additionally, the most frequent genera are *Helicobacter*, *Streptococcus* and *Prevotella*. Recent studies aimed at assessing metabolically active bacterial communities in the various gastrointestinal tracts have observed that the microbiota present in gastric juice is very similar to that found in saliva and duodenal aspirate; likewise, it appears significantly different from that found in gastric biopsies, as if the stomach had a local mucosa-associated microbiota [89]. 

The composition of the gastric microbiota depends on many different factors. Pivetta et al. [91] observed that there were differences between men’s and women’s gastric microbiota in both healthy stomachs and stomachs affected by AAG. Particularly, in healthy people, there was a lower biodiversity in females compared to that of males; instead, in patients affected by AAG, there was a significantly lower biodiversity in the males than in the females. Women with healthy stomachs had reduced biodiversity but a two-fold higher gastric bacterial abundance compared to men. On the contrary, the bacterial abundance of AAG patients was not characterized by significant gender differences, possibly because of hypochlorhydria that halved the bacterial abundance in women and had a minimum impact on it in men [91].

It has been hypothesized that a state of hypochlorhydria, as occurs in AAG, alters the gastric mucosal barrier and may promote bacterial overgrowth, thus altering the gastric microbiota [88]. In a 2017 study [92], the human gastric microbiota was evaluated in the context of hypochlorhydria caused by various factors (Hp-related CAG, AAG and PPI use); this study found that patients with AAG had a higher microbial diversity compared to patients with Hp-related gastritis (specifically, samples taken from patients with AAG showed a predominance of Streptococcus). The authors therefore concluded that the non-Hp microbiota could influence the changes in the citric acid cycle that occur in atrophic gastritis and thus may be associated with the process of gastric carcinogenesis. In a recent study [93], it was shown that the gastric microbiota in cases of corpus atrophic gastritis, predominantly negative for Hp on histological examination, exhibited higher colonization of Firmicutes, particularly Streptococcus, which were also increased in subjects with severe stages of atrophy/intestinal metaplasia at higher risk of GC according to the OLGA/OLGIM risk stratification systems. It appears interesting that streptococci, which are typically oral commensals, have also been retrieved in specimens of GC, thus raising the question of their possible role in gastric carcinogenesis; regardless, their role as transient or active resident stomach microbes still needs to be clarified [88]. A recent study showed how, compared with superficial gastritis, atrophic gastritis and intestinal metaplasia, the bacterial diversity in GC was significantly reduced, and that GC-associated bacteria (Fusobacterium, Peptostreptococcus, Streptococcus and Veillonella), highly present in tumor tissues, were prevalently distributed in microbial communities enriched by the phylum Firmicutes [94]. Thus, Firmicutes communities likely belong to the potential key bacteria for the development of GC. 

The mucosal response of chronic Hp-induced inflammation and the altered regulation of the immune response in AAG as well as the subsequent gastric dysbiosis due to the changes in the gastric microenvironment, first of all, the reduced gastric acid secretion resulting in hypo/achlorhydria, could represent one of the key factors contributing to gastric carcinogenesis by sustaining inflammation or inducing genotoxicity over time [10]. However, data in this field are scarce, and the role of the gastric microbiota in gastric carcinogenesis is still debated. Some studies have highlighted an increase in bacterial diversity in the gastric microbiota of patients with GC [45,95]; in particular, there has been an increase in oral taxa such as Streptococcus, Staphylococcus, Lactococcus, Bacillus, Prevotella, Veillonella and Leptotrichia. Furthermore, as mentioned earlier, the prevalence of streptococci, which belong to the oral microbiota, has been found in AAG. 

Therefore, the hypochlorhydric state characteristic of AAG likely promotes the growth of these bacteria, for which a role in the process of gastric carcinogenesis has been hypothesized [88]. Further studies are still needed to understand whether the microbial profile associated with hypochlorhydria conditions like AAG could be utilized in the development of GC prevention strategies.

## 7. AAG and Hypochlorhydria: Is There an Increased Risk of Gastric Neoplasms?

AAG has been associated with the development of intestinal-type gastric cancer (GC) and type 1 gastric neuroendocrine tumors (t1-gNETs). The incidence of these gastric neoplasms has been reported to be higher in patients with AAG compared to the general population (incidence ranging from 0% to 1.8% per year for intestinal adenocarcinoma and from 0.4% to 7% for t1-gNET) [96,97,98,99].

GC is globally classified as the fifth most common tumor by incidence and the fourth leading cause of cancer-related death worldwide, still representing a significant global health issue [100]. Hp infection is one of the main risk factors for GC [101]; this Gram-negative bacterium has been classified as a class 1 carcinogen by the International Agency for Research on Cancer (IARC) [102], as the chronic inflammation caused by Hp can trigger the histopathological alterations that ultimately lead to GC [103]. These alterations primarily include chronic atrophic gastritis (CAG), which, by causing a loss of glands in the gastric corpus mucosa, determines hypochlorhydria and consequently hypergastrinemia. Subsequently, CAG can lead to the development of pseudo-pyloric metaplasia, namely SPEM (which seems to protect against neoplastic development in corpus atrophic gastritis) [104] and intestinal metaplasia (IM), which predisposes a patient to the development of gastric dysplasia (also known as intraepithelial neoplasia or non-invasive neoplasia) [105,106], and consequently GC [107]. Therefore, CAG and IM represent precancerous conditions, while gastric dysplasia is a precancerous lesion [108], as exemplified by the gastric carcinogenesis sequence known as the “Correa cascade” [109]. In addition to Hp, it has long been postulated that AAG may also predispose a patient to the development of GC [10], although this is still debated. A recent meta-analysis showed an increased pooled relative risk for GC development in AAG patients (RR 11.0, 95% CI 6.49–19.1) [110]. Rugge et al. [16] concluded that in patients in whom previous or current Hp infection has been definitively ruled out (through serology, histology and molecular tests), the corpus-restricted inflammation and atrophy characteristic of AAG does not result in a significant increase in the risk of GC. In contrast, other recent prospective studies reported an increased incidence of GC and high-grade dysplasia in a long-term follow-up in Hp-negative patients with AAG [111,112]. Methodological issues and different study populations, such as studies being limited to PA or lacking exclusion of Hp infection or inclusion of patients at a pre-atrophic stage, are probably responsible for the different GC risk estimates in AAG [45,113]. In any case, this debate has raised two significant issues: the need to define precise diagnostic criteria for AAG and specific criteria for excluding current or previous Hp infection in patients with corpus-restricted CAG [113]. Further studies including AAG patients in whom previous Hp infection was accurately excluded by carefully collected medical history and histology, serology and molecular markers should help to confirm that pure AAG may have an intrinsic GC risk or whether this is due to undiagnosed previous Hp infection. 

**Figure 1:**
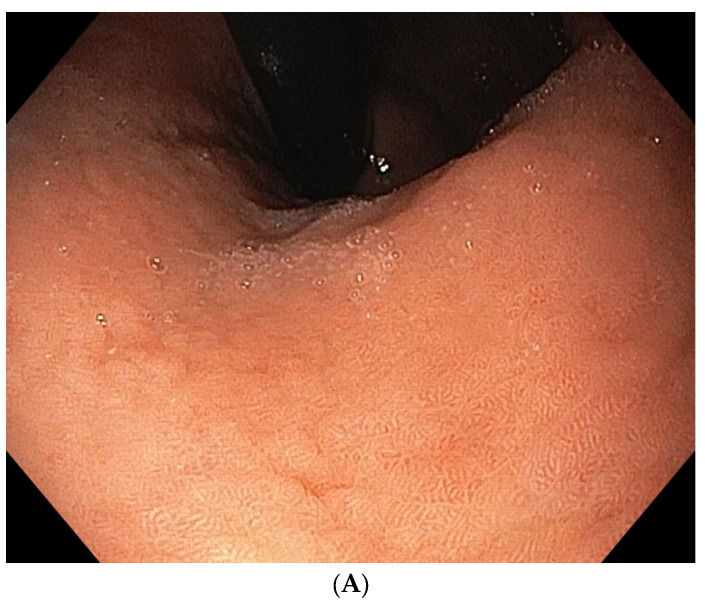

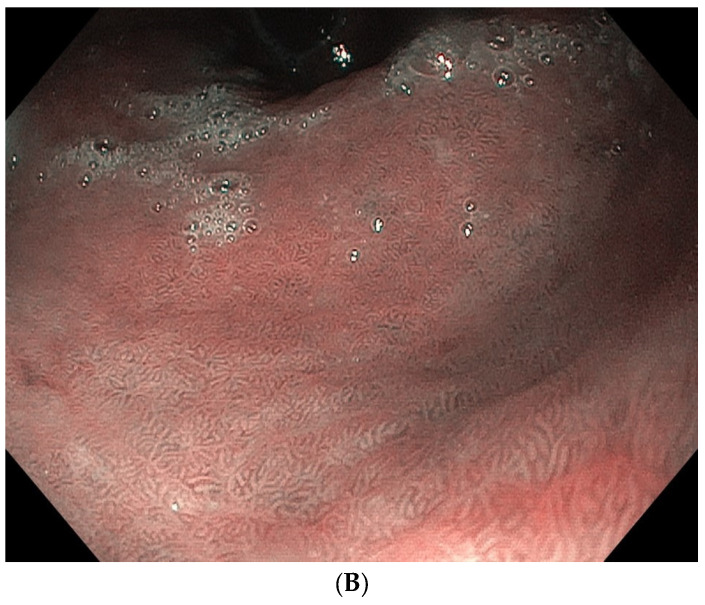
Endoscopic appearance of metaplastic autoimmune atrophic gastritis localized in the corpus mucosa in white-light endoscopy (**A**) and electronic chromoendoscopy (narrow band imaging) (**B**).

According to the latest European guidelines on the diagnosis and treatment of precancerous stomach conditions (MAPS II), AAG is a precancerous condition, and the increased risk of GC and t1-gNETs in AAG patients justifies the importance of monitoring these patients through endoscopic surveillance by gastroscopy with biopsies (according to the updated Sydney system) at intervals of 3–5 years [108], preferably with the assistance of chromoendoscopy or virtual chromoendoscopy, such as narrow-band imaging (NBI), which improves the detection of pre-neoplastic gastric conditions (such as IM) compared to traditional white-light endoscopy (WLE) (Figure 1A,B) [114,115]. It should be emphasized that the potential mechanisms of gastric carcinogenesis in AAG are currently unclear and subject to debate. In the onset of GC in AAG patients, mechanisms related to the inflammatory process, dysregulation of the host immune system and an increase in nitrose-producing bacteria due to gastric dysbiosis have been hypothesized, conditions that typically occur in case of an increased intragastric pH and hypochlorhydria associated with AAG [116,117,118]. In fact, in the context of AAG, the development of GC and t1-gNETs appears to be strongly linked to the hypochlorhydria condition due to oxyntic mucosa atrophy. When a condition of hypochlorhydria or, over time, achlorhydria develops due to the loss of oxyntic glands, a state of hypergastrinemia consequently occurs, which in turn stimulates the proliferation of enterochromaffin-like (ECL) cells. This proliferation first determines hyperplasia (linear and micronodular), which may evolve into dysplasia of the ECL cells and subsequently to the onset of t1-gNETs [119,120,121]. Waldum et al. have emphasized the role of hypergastrinemia in the onset not only of t1-gNETs but also of gastric adenocarcinomas [122,123]. 

The onset of t1-gNETs is significantly higher in the context of AAG compared to Hp gastritis, although ECL hyperplasia and t1-gNETs are not exclusive of AAG but can also occur in Hp gastritis and during long-term treatment with proton pump inhibitors (PPIs) [120,124,125]. This probably depends on the higher levels of gastrin that occur in AAG as they cause a form of corpus-restricted gastritis and the sparing of the antrum does not affect the production of gastrin by G cells. In Hp gastritis, the secretion of gastrin by G cells is partly altered by the Hp infection itself (which primarily affects the gastric antrum); a state of chronic hypergastrinemia may be present, which, analogously to AAG, stimulates ECL cell proliferation, thus possibly favoring the accumulation of mutations and increased risk of GC [122]. In AAG, the state of marked hypergastrinemia may play a role in predisposing to the development of intestinal adenocarcinoma, which has been proposed to originate from stem cells whose proliferation is stimulated directly or indirectly, through ECL cells, by gastrin [123]. A recent multicentric study describing the histopathological and molecular features of GC lesions diagnosed in AAG patients showed a high neuroendocrine component (28%) in corpus GC, supporting this idea [126]. Further research is needed to shed light on the possible carcinogenetic mechanisms in AAG.

## 8. Conclusions and Future Directions

AAG is a substantial benign condition when micronutrient deficiencies are promptly and correctly treated. The diagnosis of AAG still relies on histopathological assessment of gastric biopsies, but clinical suspicion can be supported by non-invasive serological markers such as gastrin and pepsinogens for gastric corpus atrophy and PCAs and IFAs for gastric autoimmunity, reducing the delay in diagnosis. Therefore, it is particularly important to pay attention to potential signs and symptoms of autoimmune gastritis, such as iron deficiency anemia in the absence of gastrointestinal bleeding; long-standing dyspepsia, especially when characterized by early satiation and postprandial fullness; possible clues of cobalamin deficiency such as thrombotic events, vague neurological symptoms and infertility; and the presence of other autoimmune diseases. In these cases, non-invasive serological markers may help rule out the presence of autoimmune gastritis or indicate the need for gastroscopy with biopsies. A self-administered “red flags questionnaire” comprising 25 items regarding possible symptoms or other characteristics for easing the early diagnosis of AAG has been proposed, but external validation of this tool in primary care settings is still awaited [127]. 

**Figure 2:**
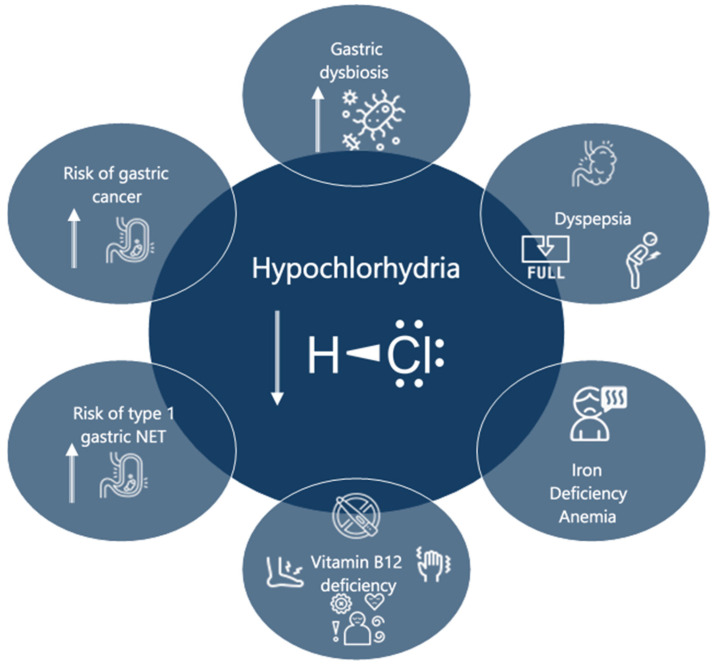
Main clinical manifestations in AAG as a consequence of impaired gastric acid secretion.

Impaired gastric acid secretion due to oxyntic mucosa atrophy leads to the main clinical manifestations and long-term complications shown in Figure 2. In particular, gastric dysbiosis is an intriguing field that can help to explain and, hopefully, prevent the increased risk of GC in AAG, but further research is needed. Other interesting topics of AAG that need to be further addressed in depth are the very early phases of the disease in which atrophy has not yet developed and which may be potentially reversible by specific treatments targeting inflammation and leading to a real change in the natural history of the disease.
